# Bioluminescence imaging of leukemia cell lines *in vitro* and in mouse xenografts: effects of monoclonal and polyclonal cell populations on intensity and kinetics of photon emission

**DOI:** 10.1186/1756-8722-6-10

**Published:** 2013-01-23

**Authors:** Sandra Christoph, Jennifer Schlegel, Francesca Alvarez-Calderon, Yong-Mi Kim, Luis N Brandao, Deborah DeRyckere, Douglas K Graham

**Affiliations:** 1 Department of Pediatrics, University of Colorado Denver, School of Medicine, 12800 E. 19th Ave., Aurora, CO, 80045, USA; 2 Department of Bone Marrow Transplantation, University Hospital of Essen, Hufelandstr. 55, Essen, 45122, Germany; 3 Integrated Department of Immunology, National Jewish Health, 1400 Jackson St, Denver, CO, 80206, USA; 4 Medical Scientist Training Program, University of Colorado Denver, School of Medicine, 12800 E. 19th Ave., Aurora, CO, 80045, USA; 5 Children’s Hospital Los Angeles, 4650 Sunset Blvd., Los Angeles, CA, 90027, USA

**Keywords:** Bioluminescence imaging, Monoclonal population, Polyclonal population, Firefly luciferase, Leukemia, Xenograft

## Abstract

**Background:**

We investigated the utility of bioluminescence imaging (BLI) using firefly luciferase in monoclonal and polyclonal populations of leukemia cells *in vitro* and *in vivo*.

**Methods:**

Monoclonal and polyclonal human lymphoid and myeloid leukemia cell lines transduced with firefly luciferase were used for BLI.

**Results:**

Kinetics and dynamics of bioluminescence signal were cell line dependent. Luciferase expression decreased significantly over time in polyclonal leukemia cells *in vitro.* Transplantation of polyclonal luciferase-tagged cells in mice resulted in inconsistent signal intensity. After selection of monoclonal cell populations, luciferase activity was stable, equal kinetic and dynamic of bioluminescence intensity and strong correlation between cell number and light emission *in vitro* were observed. We obtained an equal development of leukemia burden detected by luciferase activity in NOD-scid-gamma mice after transplantation of monoclonal populations.

**Conclusion:**

The use of monoclonal leukemia cells selected for stable and equal luciferase activity is recommended for experiments *in vitro* and xenograft mouse models. The findings are highly significant for bioluminescence imaging focused on pre-clinical drug development.

## Background

Although animal models of leukemia often utilize survival time as the primary therapeutic end point, bioluminescence imaging (BLI) is increasingly being used to provide quantitative and more rapid assessment of drug efficacy in pre-clinical oncology research [1-5]. BLI of firefly luciferase activity provides a cost-effective and extremely sensitive method for imaging fundamental biological processes *in vitro* and *in vivo*[6-8]*. In vivo* BLI is an excellent method to gain a dynamic, longitudinal profile of engraftment [9]. Luciferase oxidizes luciferin in the presence of adenosine tri-phosphate (ATP) and oxygen to form an electronically excited oxy-luciferin species. Visible light is emitted following the relaxation of excited oxy-luciferin to its ground state [10,11]. Because this light can be transmitted through mammalian tissues, it is possible to use bioluminescence for non-invasive and quantitative monitoring of leukemia burden. However, the establishment of clinically relevant animal models that include sensitive detection of early cancer growth and leukemia burden remains an ongoing challenge in translational oncology research [12]. Therefore, the difficulty in molecular imaging is in the development of effective imaging strategies with reporter systems that reveal cellular and molecular processes consistently throughout an entire study period [13-16]. Nevertheless, there are limitations associated with this approach. Using firefly luciferase as a reporter system requires exogenous luciferin addition and is currently not practical for large animal models. The rapid consumption of D-luciferin can potentially lead to an unstable signal [17]. Further mammalian tissue is known to be a turbid medium that both scatters and absorbs photons. This is mostly due to changes in refractive index at cell membranes and internal organelles, and can lead to a scattered and attenuated bioluminescence signal, which has influence on investigations especially in deeper tissue [18]. Bioluminescence imaging using firefly luciferase *in vitro* and *in vivo* is also often performed with potentially unstable luciferase-expressing polyclonal cell populations. In this study we investigated the limitations, advantages and disadvantages of bioluminescence imaging using a firefly luciferase system with monoclonal and polyclonal human leukemia cell populations *in vitro* and in a xenograft mouse model.

## Results

### Instability and incomparability of luciferase activity in polyclonal human leukemia cell lines *in vitro*

**Figure 1:**
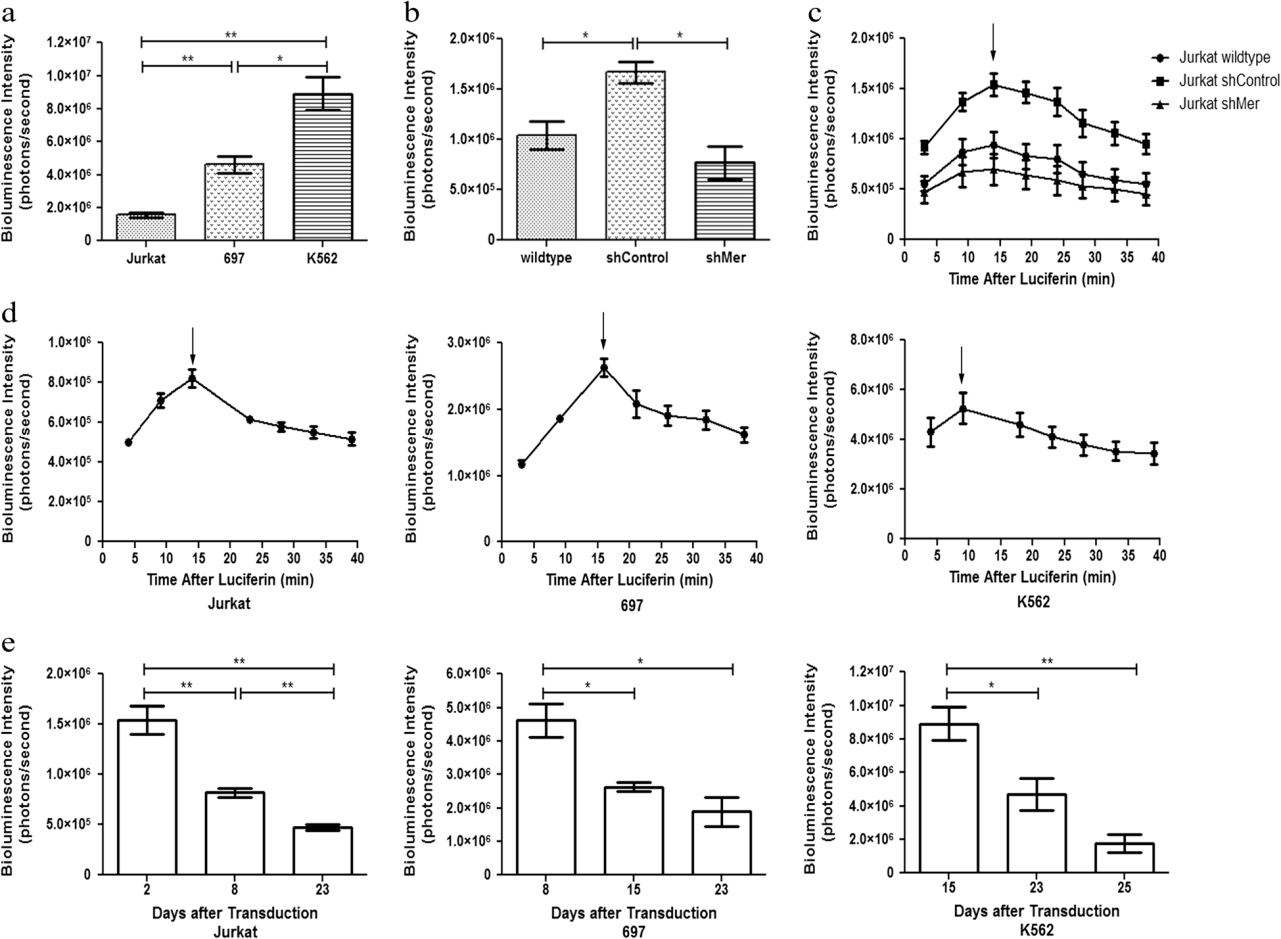
**Magnitude and kinetics of luciferase activity are cell line dependent and luciferase activity decreases over time in polyclonal cells.** Polyclonal populations of the indicated cell lines were plated (10^6^ cells/ml) and bioluminescence intensity was determined after addition of D-luciferin (150 μg/ml) to the media (Bining: 8, FOV 13.2, f/stop 1, exposure time 20 sec). Mean values and standard errors were derived from three independently transduced parental cell lines. **a-b** Maximum bioluminescence signals for the indicated cell lines. Bioluminescence images were taken 14–17 min (mean 15.6 min) after administration of D-luciferin. **c-d** Dynamic change of the bioluminescent signal in leukemia cell lines (Jurkat, 697 and K562) 8 days post-transduction and Jurkat derivatives 18 days post transduction. Signal intensities are shown as a function of time after addition of D-luciferin. **e** Bioluminescence intensities over time post-transduction are indicated. Bioluminescence images were taken 14–17 min (mean 15.6 min) after administration of D-luciferin.

Polyclonal luciferase expressing populations of human T-cell acute lymphoblastic leukemia (Jurkat), B-cell acute lymphoblastic leukemia (697) and chronic myeloid leukemia (K562) cell lines were generated and luciferase activity was determined by measurement of bioluminescence intensity (photons/second). For the Jurkat cell line and the different cell line derivatives (wildtype, shControl and shMer), equal proliferation was evaluated using cell growth curve and MTT analysis (data not shown). All cell lines were passaged twice at a density of 5 x 10^5^ cells/ml prior to transduction to ensure equal rates of cell proliferation and transductions were performed concurrently using a single aliquot and preparation of virus with the expectation of equal levels of luciferase activity. The maximum bioluminescence intensity was cell line dependent (Figure 1a). The maximum detectable bioluminescence intensity of the K562 cell line (8.92 × 10^6^ ± 0.97 photons/second) was 1.9 fold higher than the maximum signal of the 697 cell line (4.61 × 10^6^ ± 0.49 photons/second) and 5.8 fold higher than the signal of the Jurkat cells (1.54 × 10^6^ ± 0.14 photons/second). Targeted genetic modification of a single cell line, such as the use of lentivirus-mediated shRNAs to knock-down Mer receptor tyrosine kinase expression (Jurkat shMer) or as a non-silencing control (Jurkat shControl) also had a significant effect on the maximum bioluminescence signal. The signal detected in the Jurkat shControl cell line was significantly higher than the signal measured in the Jurkat wildtype or Jurkat shMer cell lines (Figure 1b).

Further, we observed a change in the kinetics of the bioluminescence signal in the leukemia cell lines (Jurkat, 697 and K562) after administration of D-luciferin. In all cell lines, the signal intensity increased to a maximum after injection of D-luciferin and then decreased slowly over time (Figure 1c, d). The kinetics of the bioluminescence signal were determined for the K562, 697 and Jurkat cell line eight days post-transduction and for the derivatives of the Jurkat cell line (wildtype, shControl and shMer) eighteen days post-transduction. The magnitude and timing of the maximum bioluminescence signal were cell line dependent. The maximum signal intensity was reached between 9 and 16 minutes after the administration of D-luciferin and was also cell line dependent (Figure 1c, d).

Finally, bioluminescence intensity decreased significantly in polyclonal luciferase-transduced leukemia cell lines over repeated passages (Figure 1e). The rate of luciferase signal decay was also cell line dependent, but all 3 cell lines exhibited significantly decreased signal intensity (>50%) within 3–4 weeks after transduction. The reduction was most dramatic in K562 cells, where bioluminescence intensity was decreased 80.4% at 25 days post transduction relative to the initial signal.

### Inconsistent luciferase activity in a xenograft mouse model after transplantation of polyclonal leukemia cell lines

**Figure 2:**
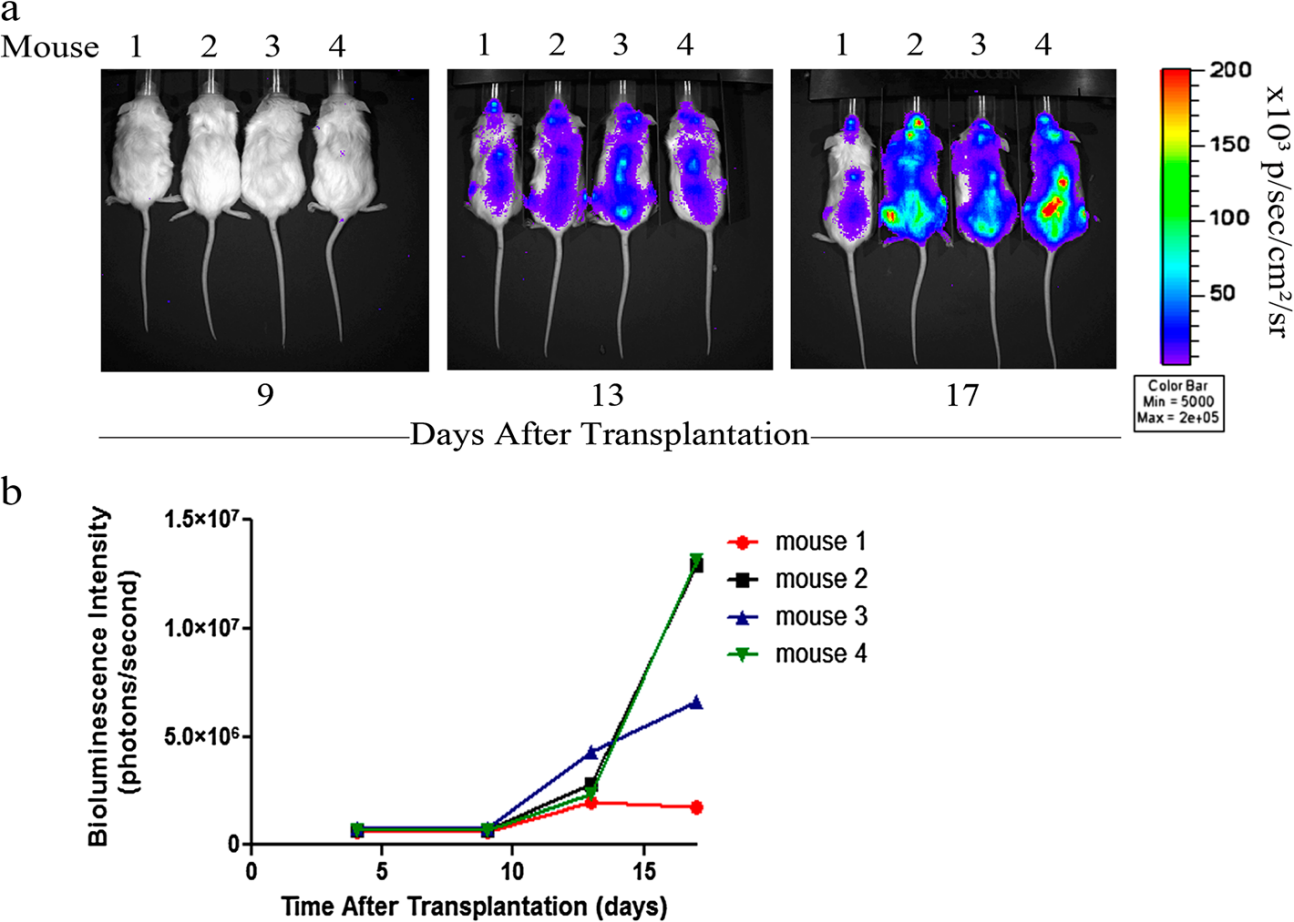
**Longitudinal quantification of bioluminescent signals in mice transplanted with polyclonal luciferase-transduced human leukemia cell lines.** Four NSG mice were transplanted with 5 x 10^6^ polyclonal luciferase-transduced Jurkat wildtype cells by intravenous injection into the tail vein. D-luciferin was injected intraperitoneally (150 mg/kg body weight) and bioluminescence images were taken twice weekly (Bining: 8, FOV 19.6, f/stop 1, exposure time 120 sec). **a** Pseudocolor images of mice transplanted with polyclonal luciferase-expressing human Jurkat wildtype cells showing unequal development of bioluminescence signal over the time. No luciferase signal was detected in control mice (data not shown). **b** Signal intensity was quantified from each animal plotted and is shown as a function of the number of days after transplantation.

A xenograft mouse model was utilized to determine if the selective pressure against luciferase activity was also present *in vivo* and to investigate the consistency of luciferase activity in polyclonal luciferase-transduced leukemia cell lines *in vivo*. Sub-lethally irradiated NOD scid gamma (NSG) mice were transplanted with polyclonal luciferase-expressing Jurkat cells via tail vain injection and *in vivo* BLI was performed. Light emission was first detected on the third day after transplantation of the cells. During the test period of 17 days, light emission was evident throughout the body (Figure 2a) indicating diffuse distribution of the injected cells. Relatively strong signals were observed in spine, head, and femur. There was no light emission detected in the control groups, which were transplanted with non-transduced Jurkat cells or mock-transplanted with PBS (data not shown). The bioluminescence signals observed for mice transplanted with a polyclonal population of luciferase-transduced Jurkat cells varied greatly. After 17 days the bioluminescence intensity ranged from 1.8 × 10^6^ photons/second in mouse 1 to 13 × 10^6^ photons/second in mouse 2 and 4, equivalent to a greater than 7-fold difference in bioluminescence intensity (Figure 2b).

### Stability and dynamics of luciferase activity in monoclonal human leukemia cell lines *in vitro* and in a xenograft mouse model

**Figure 3:**
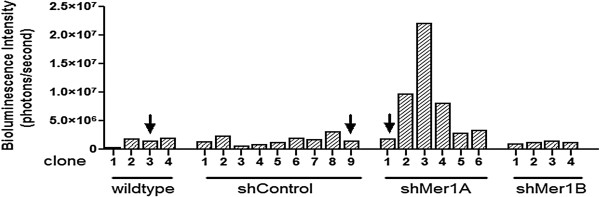
**Generation of monoclonal luciferase-transduced human leukemia cell lines.** Monoclonal luciferase-transduced Jurkat cell lines (wildtype, shControl, shMer1A and shMer1B) were isolated and bioluminescence intensity was determined as an indicator of luciferase activity (Bining: 8, FOV 13.2, f/stop 1, exposure time 5 sec). Four Jurkat wildtype clones, nine shControl clones, six shMer1A clones and four shMer1B clones were developed and analyzed. Jurkat wildtype clone 3, shControl clone 9 and shMer1A clone 1 had equal levels of luciferase activity and were selected and used for further studies.

To study the effects of an exclusively monoclonal population on the stability and comparability of luciferase activity as detected by bioluminescence intensity *in vitro* and *in vivo*, we generated monoclonal luciferase-transduced cell populations from single cells by sorting via flow cytometry. After confirmation of suitable growth and viability, the 697 and K562 clones with the strongest bioluminescence signal and luciferase activity were chosen for further investigation. For the Jurkat cell line derivatives expressing shRNA, one clone of each cell line derivative was chosen so that the luciferase activity within the panel of cell lines was similar (Figure 3). Because the Jurkat shMer1A clone had a greater knockdown of the Mer receptor tyrosine kinase expression, the Jurkat shMer1B clone was not used for further analyses (data not shown).

**Figure 4:**
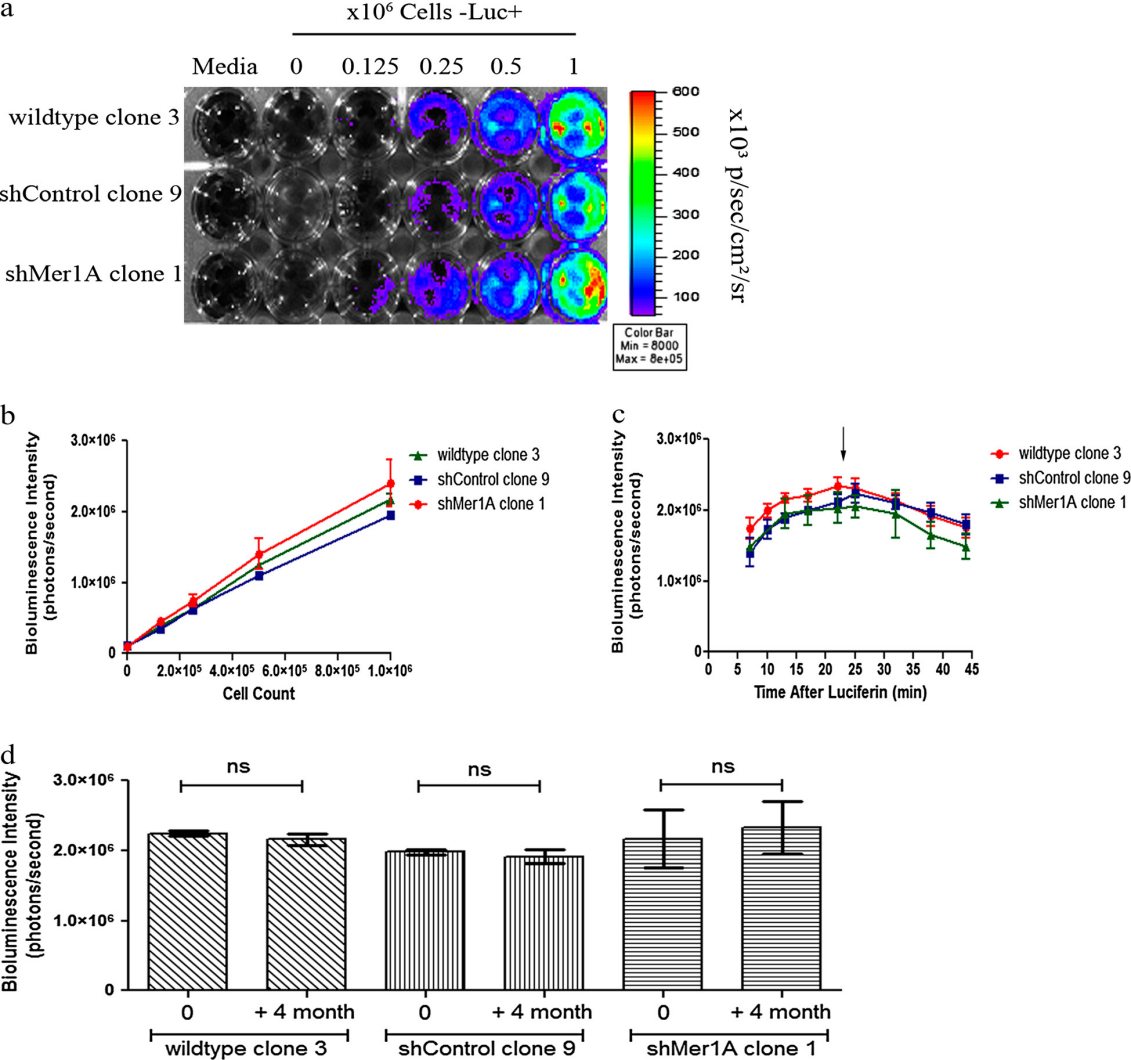
***In vitro *quantitation of bioluminescence signal in monoclonal human leukemia cell lines expressing luciferase.a** Pseudocolor representation of the bioluminescence intensity from monoclonal luciferase-transduced Jurkat cell lines (wildtype, shControl, shMer1A). Cell concentrations ranging from 1.25 x 10^5^ to 1 x 10^6^ cells were plated in a 24-well plate and images were captured after addition of D-luciferin to the media. Wells containing medium only with or without D-luciferin served as negative controls. **b** Correlation between cell number per well and bioluminescence intensity (photons/second per well) for three cell line derivatives. Mean values (+/− SEM) were determined from three separate experiments. The measured intensity of bioluminescence was directly proportional to the number of cells. **c** Bioluminescence intensity as a function of time after luciferase addition in monoclonal luciferase-transduced Jurkat cell lines (wildtype, shControl, shMer1A). Mean values and standard errors (+/− SEM) were derived from three independent experiments. No significant differences in the dynamics of signal intensity over time were observed for the selected clones. **d** Stability of luciferase activity of three monoclonal populations of the Jurkat cell line (wildtype, shControl, shMer1A). Cells were passaged for four months and luciferase activity was monitored by measurement of bioluminescence intensity. Mean values (+/− SEM) derived from three independent measurements. All clones exhibited stable luciferase activity throughout the test period.

To assess the relationship between bioluminescence signal intensity and viable cell numbers of a monoclonal luciferase-transduced cell population *in vitro*, we prepared a dilution series of monoclonal luciferase-transduced Jurkat wildtype, Jurkat shControl and Jurkat shMer1A cells (range 1.25 × 10^5^ - 1 x 10^6^ cells/ml) and measured the bioluminescence intensity for a given volume of cell suspension. The bioluminescence intensity increased proportionally with increasing cell numbers (Figure 4a, b). A strong correlation between number of cells and light emission was obtained (R^2^_wildtype clone 3_ = 0.99, R^2^_shControl clone 9_ = 0.99, R^2^_shMer1A clone 1_ = 0.90). Further, we saw similar kinetics of the bioluminescence signal in Jurkat cell lines (Jurkat wildtype, Jurkat shControl and Jurkat shMer1A cells) after administration of D-luciferin. The bioluminescence intensity gradually increased to a maximum, then decayed over time. Maximum bioluminescence intensity, signal increase, and time of peak were similar for the different cell line derivatives (Figure 4c). In order to assess the stability of luciferase activity over an extended period of time, the monoclonal Jurkat populations were cultured for 4 months (> 30 passages). For all three selected Jurkat cell clones, we confirmed stability of bioluminescence intensity over extended passages in monoclonal populations (Figure 4d).

**Figure 5:**
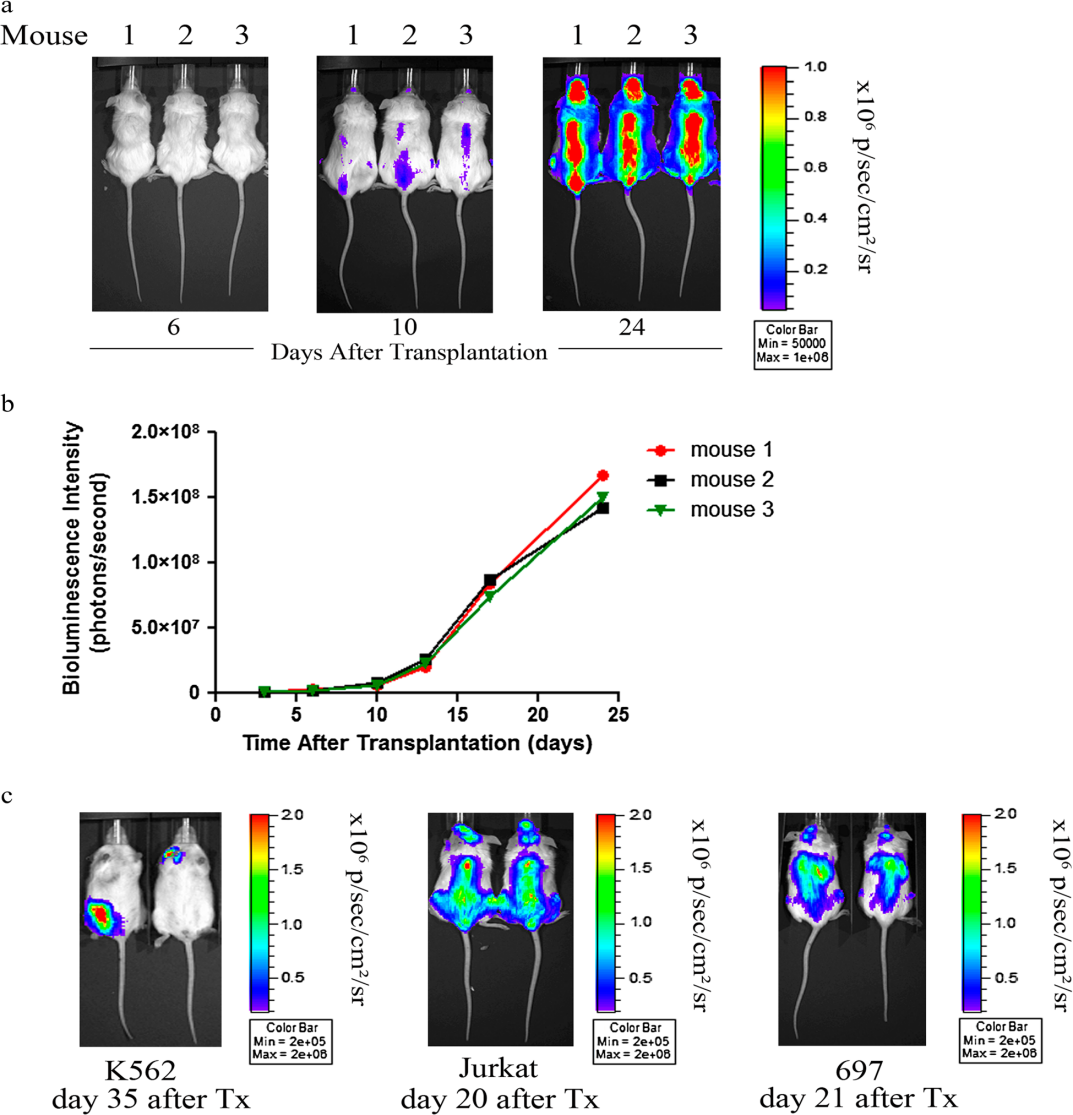
**Longitudinal quantitation of bioluminescence signals in mice transplanted with monoclonal luciferase-transduced human leukemia cell lines. a** NSG mice were transplanted with 5 x 10^6^ monoclonal luciferase-transduced wildtype Jurkat cells by injection into the tail vein. D-luciferin was injected intraperitoneally and bioluminescence images were taken twice weekly (Bining: 8, FOV 19.6, f/stop 1, exposure time 120 sec). Pseudocolor images of mice transplanted with monoclonal luciferase-expressing human Jurkat wildtype cells showing equal development of bioluminescence intensity over time. **b** Quantitation of bioluminescence signal from each animal plotted against the number of days after transplantation. **c** Pseudocolor images of two representative mice transplanted with monoclonal luciferase-expressing human leukemia cells (Jurkat, 697 and K562) showing equal development of bioluminescence intensity and demonstrating establishment of leukemia in different organs.

Monoclonal luciferase-expressing Jurkat wildtype cells were injected via tail vein into sub-lethally irradiated NSG mice and *in vivo* bioluminescence imaging was performed in an attempt to examine the consistency of luciferase activity in monoclonal luciferase-transduced leukemia cell lines *in vivo.* Importantly, we found an equal and comparable development of bioluminescence signal after transplantation of monoclonal luciferase expressing cell lines (Figure 5a, b). Equal and comparable development of bioluminescence signal was also noted after transplantation of monoclonal populations of luciferase-transduced 697 cell lines in NSG mice (Figure 5c). In addition, knowledge was gained regarding early anatomic localization of engraftment and organ specific homing of different leukemia entities (T-ALL, B-ALL and CML) in NSG mice (Figure 5c). Throughout the course of imaging, the strongest bioluminescence signals appeared in the vertebral column, pelvis, and femurs after transplantation of luciferase-transduced Jurkat cells. After transplantation of luciferase-transduced 697 cells, we measured the strongest bioluminescence signal in the liver and the femurs. The strongest bioluminescence signal after transplantation of luciferase-transduced K562 cells was seen in the lymph nodes. The extent of leukemic infiltration of different organs was cell line dependent.

## Discussion

To the best of our knowledge, this study is the first reported investigation comparing bioluminescence imaging using a firefly luciferase system in monoclonal and polyclonal human leukemia cell populations *in vitro* and in a xenograft mouse model. We have shown that the bioluminescence signal intensity and the dynamics of luciferase activity *in vitro* were cell line dependent (Figure 1). Moreover, bioluminescence signal intensity was unstable in polyclonal populations and decreased significantly with repeated passage in culture. The decreasing signal observed may be due to survival and growth advantages for the non-transduced cells within the polyclonal cell population. We also observed dramatic variability in signal intensity in sub-lethally irradiated NSG mice transplanted with a polyclonal luciferase-transduced cell population, suggesting that in a polyclonal population, different clones contribute to establishment of disease in different mice (Figure 2). This source of heterogeneity significantly decreases the power of this model system to determine differences in disease progression related to experimental treatments. In addition, the variability in bioluminescence signal we observed in polyclonal luciferase-transduced populations limits side-by-side comparison of cell line derivatives with targeted genetic manipulations, where differences in signal intensity between cell lines will optimally be due solely to differences in disease progression, rather than cell line dependent differences in transduction efficiency and/or luciferase activity.

With the generation of monoclonal luciferase-expressing leukemia cell lines, stability of luciferase activity over a long period of time (> 4 months) was obtained. Comparable bioluminescence intensity in monoclonal luciferase-tagged cell lines with targeted genetic modifications was also observed. Moreover, monoclonal cell populations showed the same dynamic of bioluminescence intensity after administration of D-luciferin *in vitro,* revealing a significant advantage of this method (Figure 4). Most importantly, transplantation of monoclonal luciferase-transduced cell lines in a xenograft mouse model resulted in genetically and phenotypically identical disease with comparable disease kinetics, thereby significantly improving the utility and sensitivity of this model (Figure 5).

In the system used for our investigations, we demonstrated that the selection of monoclonal luciferase-expressing populations based on equal luciferase activity resulted in isolation of cell lines that were directly comparable, both *in vitro* and *in vivo*. Commonly the use of an antibiotic resistance marker would likely be sufficient for *in vitro* models. However, we avoided the treatment of leukemia cells with additional antibiotic agents to minimize putative *in vivo* drug interactions in future translational drug studies. In addition, antibiotic selected cell populations maintain a heterogeneous expression of luciferase, which may introduce variability to the transplanted cell population and may affect the consistency during the experiment.

The method to transplant monoclonal luciferase-transduced cell populations in murine xenograft models presented here has several advantages, but there are also some limitations which should be mentioned. First, we have seen that survival in xenograft models can be impacted by lentivirus transduction and/or luciferase expression in leukaemia cells and thus, these models may less accurately represent the normal biology of leukemogenesis (data not shown). Second, our data indicate that only monoclonal populations with the same growth characteristics, luciferase activity and phenotype can be directly compared in luciferase-based murine xenograft models, limiting the ease of their utility for comparison of genetically modified cell lines and their parental counterparts.

## Conclusions

In conclusion, our data demonstrate that derivation of monoclonal cell lines is critical for development of robust, sensitive, and reproducible luciferase-based murine xenograft models. Non-invasive, longitudinal monitoring of leukemia progression in murine xenograft models based on bioluminescence intensity, as described here, will expedite the investigation and discovery of novel therapies. Using this methodology, direct comparison of leukemogenesis after targeted genetic modifications and sensitive, longitudinal determination of leukemia burden are both possible, thereby facilitating both target validation studies and robust testing of translational agents. Ultimately this approach may also prevent mischaracterization of therapies as ineffective based on unequal development of leukemia burden detected by bioluminescence intensity with the use of a polyclonal luciferase-expressing leukemia cell population.

## Materials and methods

### Cell lines

Jurkat, 697 and K562 human leukemia cell lines were obtained from the American Type Culture Collection (ATCC, Manassas, VA). All cell lines were cultured in RPMI-1640 medium (Hyclone Laboratories, Logan, UT) supplemented with 10% fetal bovine serum (FBS, Atlanta Biologicals, Lawrenceville, GA) and penicillin/streptomycin (100 units/ml and 100 μg/ml, Hyclone Laboratories, Logan, UT). Cells were maintained at 37°C in a humidified atmosphere containing 5% CO_2_. The identities of Jurkat, 697 and K562 cell lines were confirmed by short tandem repeat analysis and all cell cultures were determined to be free of mycoplasma contamination.

### Lentivirus production and cell transduction

pCCL-MNDU3-LUC is a third generation HIV-1 based, lentiviral vector containing the firefly luciferase gene (gift from Yong-Mi Kim, Children’s Hospital Los Angeles, CA) [19]. 293FT cells were transiently transfected with pCCL-MNDU3-LUC and a three-plasmid packaging system (Gag-Pol, Rev and VSV-G) using Turbofect (Fermentas, Glen Burnie, MD). Viral supernatants were harvested at 24 and 48 hours post-transfection and concentrated by ultracentrifugation. Jurkat wildtype, Jurkat shControl, Jurkat shMer1A, Jurkat shMer1B, 697 and K562 cells were transduced in the presence of polybrene (Millipore, Billerica, MA) and single cell sorting was performed using flow cytometry. Clonal populations were screened for luciferase activity by measurement of bioluminescence intensity as described below. Jurkat shControl, shMer1A, and shMer1B cell lines were generated using lentiviral shRNA vectors shMer1 (targeting Mer) and shControl (a non-silencing vector ) as previously described [20].

### *In vitro* bioluminescence imaging

Actively growing human leukemia cells expressing luciferase were harvested from 10 cm^2^ tissue culture plates and viable cell counts were via trypan blue dye exclusion staining determined as the average of two counts using a hemocytometer. Serial dilutions of cells ranging from 1.25 × 10^5^ to 1 × 10^6^ cells per well were plated in 1 ml of medium in 24-well tissue culture plates. Untransfected human leukemia cells were plated in the same manner to determine auto-fluorescence for each population size. Wells containing medium only were used to detect background fluorescence. D-luciferin (Caliper Life Sciences, Hopkinton, MA) was added to a final concentration of 150 μg/ml immediately before bioluminescence imaging. Photon counts per second were recorded using an IVIS200 (Xenogen, Alameda, CA) imaging system and analyzed with Living Image 3.2 software (Caliper Life Sciences, Hopkinton, MA). Changes in bioluminescence intensity over time were measured and are presented as total flux values in photons/second for each well. Reported results are the average of three independent experiments.

### Murine xenograft model

NOD scid gamma mice (NSG, Stock # 5557, The Jackson Laboratory, Bar Harbor, ME) were sub-lethally irradiated with 200 rads and intravenously transplanted with luciferase-expressing human leukemia cells (5 × 10^6^ cells). Transplanted mice underwent *in vivo* bioluminescence imaging at various times as specified for each experiment. Animals were monitored daily and were euthanized upon signs of leukemia onset (weight loss >15%, decreased activity, and/or hind limb paralysis). All experiments involving animals followed the regulatory standards approved by the University of Colorado Institutional Animal Care and Use Committee.

### *In vivo* bioluminescence imaging

NSG mice were anesthetized with inhaled isoflurane and were maintained with 1.5-2% isofluorane during imaging procedures. Luciferase-based bioluminescence imaging was performed with an IVIS200 imaging system equipped with a camera box and warming stage. Following intraperitoneal injection of 150 mg/kg D-luciferin dissolved in phosphate buffered saline (PBS), mice were immediately imaged with sequential 30, 60, 90 and 120 seconds exposures. Images were captured and bioluminescence intensity was quantitated using Living Image 3.2 acquisition and analysis software (Caliper Life Sciences, Hopkinton, MA). Total flux values were determined by drawing regions of interest (ROI) of identical size over each mouse and are presented in photons (p)/second (sec).

### Statistical analyses

The mean bioluminescence intensities (photons/second) and corresponding standard errors were determined for each experiment. For all measurements, data are presented as mean ± standard error of the mean (SEM). The student’s t test was used to determine the significance of differences between means. The level of significance for all statistical analyses was chosen a priori to be p < 0.05. Statistical analyses were carried out using Prism software (Version 5.0, GraphPad Software, LaJolla, CA).

## Competing interest

The authors declare that they have no competing interests.

## Authors’ contributions

SC designed methods and experiments, carried out the experiments, analyzed the data, interpreted the results and wrote the paper. JS contributed to the study design and the data interpretation. FAC contributed to the data collection. YMK generated the lentiviral vector containing the firefly luciferase gene. LNB generated the Jurkat shControl and shMer knockdown cell line. DD contributed to the study design and the data interpretation. DKG contributed to the study design and the data interpretation. All authors read and approved the final manuscript.
